# Supplementary material for: A cautionary note on the use of N-acetylcysteine as a reactive oxygen species antagonist to assess copper mediated cell death
Source: PLoS One. 2023 Dec 11;18(12):e0294297. doi: 10.1371/journal.pone.0294297 (PMC10712875; doi:10.1371/journal.pone.0294297)
Supplement: Attachment — Submitted filename: response-to-reviewers.docx [file pone.0294297.s002.docx]

Associate Director, Cancer Research UK Scotland Centre

Institute of Genetics and Cancer

The University of Edinburgh

Crewe Road South

Edinburgh EH4 2XR

Telephone	+ 44	(0) 131 651 8671

Fax + 44 (0) 131 651 8711

PA: Mrs Jan Irvine– [jan.irvine@ed.ac.uk](mailto:jan.irvine@ed.ac.uk)

E-mail: [n.carragher@ed.ac.uk](mailto:n.carragher@ed.ac.uk)

October 17^th^, 2023

Dear PLOS One Editors,

We thank the editor and reviewers for their many positive comments and constructive suggestions on our submitted manuscript; ***“A cautionary note on the use of N-acetylcysteine as a reactive oxygen species antagonist to assess copper mediated cell death” [PONE-D-23-23834]*** for consideration for publication in PLOS One.

We have carefully gone through and addressed all of the editorial and reviewer’s comments in our revised version as outlined point-by-point below. In addition, we have attempted to explain some misunderstanding with regards to reviewer 1 comments 1, 2 and 8 and request further clarification with regards to reviewer 1 comments 2 and 9.

*Additional Editor Comments:*

*In my opinion, the work provides an advance towards the current knowledge. The quality and content meet standards set forth by PLOS One journal and I suggest publishing the paper in the journal with minor changes and after addressing the points raised by the reviewers. Below are some suggestions that confirm reviewers' concerns, and it is important that the authors address them with attention:*

*- Keywords: Keywords enable authors to extend the representation of their manuscript content beyond that presented in the title and abstract. Keywords make your paper searchable and ensure that you get more citations. Thus, it is important to include the most relevant keywords that will help other authors find your paper. It is therefore very important to avoid repeating keywords already cited in the title to increase the discoverability of your paper. Please revise and address.*

We thank the editor for the suggestion and have now added the following keywords:

Cuproptosis, Elesclomol, Disulfiram, N-acetylcysteine, Reactive Oxygen Species

*- The depiction of the research necessitates further elaboration. It is imperative to furnish additional details pertaining to the study both in the introduction and the discussion sections. Please elaborate and confirm with justified references.*

In line with the editor and reviewer comments we have now expanded and elaborated upon out text in the introduction and discussion to place our work into context of relevant published literature, citing additional references. We have also provided a new figure 1 to illustrate the main message of our study.

- Please revise your references section and update it as much as possible. Some interesting recent references are missing.

We have now significantly revised our reference section and updated with several new references (increasing from 21 to 41 cited references) including those recommended by both reviewers.

*Reviewer 1 comments*

*1. Section Introduction: Surprisingly, the claim of the authors on line 59-60 is not supported by corresponding citations, although it is absolutely essential for this MS! Authors should pay careful attention to this part of the argument. For example, the references 4, 8, and 9 listed do not support your claim in any way!*

We apologise for not adding citations on line 59-60. Several new references have now been added to this section (References 12,14,15,16,17,18,19) and we further elaborate on known mechanism of NAC in the following sentences citing relevant literature (references 20,21,22,23, 24 and 25). Regarding original references 4, 8 and 9, we believe there is a misunderstanding of our text. Our intention is to not make any controversial claim but present a clear fact that many publications are making overt statements that cell death in response to intracellular copper accumulation, following treatment with the copper chelator Eleseclomol, is mediated via the production of reactive oxygen species (ROS). This is exemplified in

- Original reference 4 (Introduction paragraph 2): “Elesclomol is a novel drug candidate that exerts anticancer activity through the elevation of ROS levels leading to subsequent activation of apoptosis”.
- Original reference 8 (in the title): “Elesclomol induces cancer cell apoptosis through oxidative stress”
- Original reference 9 (in the abstract): “Furthermore, treating metastatic melanoma cells with the drug, Elesclomol, which induces cancer cell apoptosis through oxidative stress…”,

We have attempted to clarify this misunderstanding by changing the text on line 60-72 to more clearly reflect that we are referencing common statements made by others in the literature on the mechanism-of-action of elesclomol.

*2. Section Introduction: Likewise, the authors' claims on lines 60-64 are not even supported by relevant citations, or even true! There are many publications on the actual (not those who are mistakenly believed) properties of NAC, the most recent comprehensive article on the subject can be found here, for example: Mlejnek P. Antioxidants (Basel). 2022, 11(8): 1485. doi: 10.3390/antiox11081485.*

We thank the reviewer for highlighting the recent review article by Mlejnek et al., 2022. This is an important review which supports our view that conclusions drawn from studies demonstrating the protective effect of NAC upon cell death is mediated by an oxidative stress-related mechanism is misleading. The review article discusses alternative mechanisms of NAC in depth but importantly does not address the effect of NAC on copper accumulation and cell death mediated via cuproptosis which we describe in our manuscript. We note the Mlejnek article also cites reference 11 (Mi L, et al., Anal Biochem. 2010;405: 269–271. doi:10.1016/j.ab.2010.06.015) that we cite here and thus we ask the reviewer for clarification if this is indeed a relevant reference? Our intention here is to highlight that NAC can influence cellular phenotypes via several ROS-dependent and ROS-independent mechanisms, however we do accept the reviewers suggestions and have substantially rewritten this section to ensure it reads as we intended (this is in agreement with, and our study provides further evidence to support, the overarching conclusions from the Mlejnek review) and we have cited more relevant literature (including the Mlejnek review) on the well-established properties of NAC.

*3. Section Materials and Methods: Unfortunately, there is no description of the cell survival assay.*

We have changed the title of the methods section from “N-acetyl cysteine dose response” to “Cell survival assays” and added subsections for each set of experiments to make this clearer. Further we apologise for not specifically mentioning the cell survival assay end point for the NAC assay and these details have now been added.

*4. Section Results: The results presented in Figures 1 and 2 are trivial and, as the authors themselves state, are in agreement with the results of other authors. Therefore, it would be more appropriate to present them more economically, e.g. in the form of one table with the relevant IC50 values or only as one Figure!*

We have now condensed all the dose responses into one figure. We believe that a dose response curve provides much more detail and information than an IC50 value so have opted for a single figure over a table.

*5. Section Results: The assertion on lines 132-134 is not justified. In reference 13 you mentioned, the authors only speculate…*

We believe our data demonstrating that NAC only prevents cell death induced by elesclomol (and disulfiram) when used at milimolar concentrations is important to highlight to the readers as this observation should serve as a warning to researchers to consider that NAC protective effect on cell death may be mediated via mechanisms which are independent or in addition to oxidative stress. We have adjusted the text to highlight that this is speculative and included the recommended review by Mlejnek which states that NAC stimulates GSH synthesis at submillimolar concentrations.

*6. Section Results: In contrast, the key claim that NAC at high concentrations prevents the "uptake" of copper into cells is very poorly (!) supported experimentally:*

*a) It is shown only for elesclomol but not for disulfiram.*

We have now performed the experiment for disulfiram in addition to elesclomol and include the results in figure 3

*b) It is shown only for one cell line.*

We have now performed the experiment across two sensitive cell lines OAC-P4C and SK-GT-4 and include the data in figure 3

*c) It is shown only for high NAC concentration.*

We now provide data across two concentrations of NAC (1mM and 10mM) across both cell lines and drugs (figure 3).

*d) There is no statistical evaluation of the results.*

We apologise for this oversight and have now added statistics (Student’s T-test) and P-values to the plot. We have added significance tests for each treatment compared to DMSO vehicle control for each cell line.

*e) Figure 3 in this MS resembles Figure 4 from the work by Hughes et al., ACS Chem Biol. 2022, 17(7): 1876-1889. doi: 10.1021/acschembio.2c00301.*

The entire ICP-MS dataset presented in the revised manuscript is new in order to add in the extra cell lines, drugs and NAC concentrations as requested by the reviewer (data generated in September 2023). We have previously published ICP-MS data to demonstrate that Elesclomol and Disulfiram both cause an accumulation of copper (doi: 10.1021/acschembio.2c00301.), but this publication did not include the NAC data critical to this publication.

*7. Section Results: It would be appropriate to show:*

*a) what ROS species are produced in your experimental system;*

*b) to find out whether their real scavengers, which do not simultaneously form chelates with copper, have an effect on survival or not. You can also find an overview of antioxidants and their properties in the above reference.*

The mechanisms by which copper chelators induce cancer cell cytotoxicty are still poorly understood and are likely to involve multiple mechanisms which may be cell line and context specific. We believe the reviewers suggestions represent interesting future studies to perform in significantly more detailed investigations on the role of copper chelators and ROS in oesophageal cancer cell cytotoxicity. However, such studies are out of scope for our current article which is focussed on the observation that NAC prevents copper accumulation and the impact this has on the utility and interpretation of NAC effects in studies of copper mediated cell death. We do now include a section in our discussion on future work including exploring additional ROS scavengers.

*8. Section Results. Without the careful analysis suggested in point 7 (see above), the results presented in Figure 5 do not make much sense.*

We disagree with the reviewers comment that the “results presented in Figure 5 do not make sense” as this is real experimental data comparing the rank order of oesophageal cancer cell sensitivity following dose-response treatments with the copper chelator compounds elesclomol and disulfiram and a well-established inducer of ROS induced cell apoptosis (H202). The results clearly demonstrate that the sensitivity profile of the oesophageal cancer cell line cytotoxicity to the copper chelators is distinct from that of a well-known inducer of ROS and as such this data stands on its own. Without speculating further on induction of different types of ROS species and antioxidant scavenger mechanisms the result indicates that oesophageal cancer cell lines display distinct sensitivity to copper chelators and this differential sensitivity cannot be mimicked by direct induction of H2O2 alone. However, we acknowledge that an extensive assessment of sensitivity to different ROS species would make an excellent future study.

*9. The Discussion section needs a very thorough overhaul.*

We have substantially re-written the discussion to include further references which place our observations and conclusions into context with the published literature. We are happy to incorporate further specific recommendations if the reviewer can clarify what they would like to see here.

*Reviewer 2 comments*

*This is an interesting study that cautions against using N-acetyl cysteine in studies on Cu mediated cell death. The manuscript can be accepted with minor revision.*

*1. The introduction needs more references to support their presumptions*

We apologise for not comprehensively citing the literature. This has now been amended.

*2. The discussion section may benefit if the work of Hadi et al. on copper and polyphenol mediated cell death is also cited; in addition to other recent references.*

We thank the reviewer for bringing this work to our attention. We have now included a brief section on other copper related drug mechanisms in the discussion.

*3. Other ROS scavengers can perhaps also be discussed in some depth.*

We have added a section to our discussion pertaining to future work with ROS scavengers, though we have included a warning as to the possibility that these too may interfere with the copper directly and this must be confirmed before conclusions are drawn as to the findings.

*4. A schematic diagram will make the paper even easier to understand than it already is.*

We thank the reviewer for this idea and agree this will help with understanding and we have now included a schematic diagram (fig 1).

*5. Can other statistical methods be used to compare and contrast results?*

We apologise for not including stats for the ICP-MS data. This has been corrected.

As instructed we have submitted a marked-up copy of our manuscript that highlights changes made to the original version and an unmarked version without tracked changes. We believe the reviewers comments and the changes we have made in response have significantly improved our manuscript. We anticipate that our manuscript, if accepted, will provide an important contribution and service to a broad research community studying ROS mediated cellular cytotoxicity and the emerging field of cuproptosis were inappropriate conclusions from experiments utilizing N-acetylcysteine are being made. We hope our revised submission satisfies both you and the reviewers and we look forward to receiving the reviewers and editorial response on our revised submission.

Yours sincerely,

Professor Neil Carragher

Personal Chair of Drug Discovery

Director of Translation

University of Edinburgh

Web; <https://www.ed.ac.uk/cancer-centre/research/carragher-group>
